# The impact of kinesiotaping on functional respiratory outcome in chest injuries—a pilot randomized trial

**DOI:** 10.7717/peerj.21753

**Published:** 2026-10-12

**Authors:** Marko Žličar, Maja Frangež, Matej Jenko, Maja Marija Potočnik, Luka Hodnik, Tajda Doupona, Alenka Spindler Vesel

**Affiliations:** 1 University Medical Centre Ljubljana, Ljubljana, Slovenia; 2 Medical Faculty, University of Ljubljana, Ljubljana, Slovenia

**Keywords:** Kinesiotaping, ELT, Chest injury, Rib fracture, Functional respiratory improvement, Pain

## Abstract

**Background:**

Kinesiotaping (KT) is used as adjunctive therapy for musculoskeletal injuries to relieve pain, improve blood and lymphatic circulation and muscle and fascial function, provide joint support, and promote proprioception.

**Aim:**

In this pilot randomized trial, we compared the differences between a group that received KT in addition to standard respiratory physiotherapy and a group that received standard respiratory physiotherapy only. We examined changes in pain intensity and functional respiratory parameters at study entry and on the first day after study enrolment and assessed the functional status of patients 10 days after study enrolment.

**Methods:**

Thirty-four patients with isolated unilateral rib injuries (less than five ribs) participated in the study. Patients were randomly divided into two groups. In the study group (SG), KT was applied, and both SG and the control group (CG) performed breathing exercises. Spirometry was used to measure functional respiratory parameters and visual analogue scale (VAS) and algometry were used to measure pain intensity immediately after the application of KT and breathing exercises/breathing exercises only (in SG and CG respectively) and on the day after enrolment in both groups. The functional status of patients was assessed 10 days after enrolment using EQ-5D questionnaire.

**Results:**

There was no improvement in respiratory parameters between groups. There was no influence of KT on pain, both measured with VAS and algometry. Variance analysis suggests a possibility of good and poor responders to KT.

**Conclusions:**

The use of KT in patients with isolated unilateral rib injuries in this pilot study had no effect on functional respiratory parameters nor on perception of pain.

## Introduction

Chest injuries are the third most prevalent injury worldwide, following only head trauma and injuries to the extremities. Chest injuries can be divided into penetrating injuries and blunt trauma, based on the mechanism of injuries. Penetrating injuries, such as stabbing, cutting and firearm injuries, are disruptive to tissue integrity (Ludwig & Koryllos, 2017; Adal et al., 2024). Blunt chest trauma accounts for 15% of all trauma cases in the world. Blunt injuries are usually the result of traffic accidents, falls from height, occupational accidents or suffered during sport activities and represent 70% of all chest trauma (Dogrul et al., 2020). The age-standardized incidence rate of severe chest injury was 102 cases per 100,000 individuals, and the prevalence rate was 25 cases per 100,000 individuals globally. Central Europe, which includes Slovenia, exhibits some of the highest incidence and prevalence rates globally (age-standardized incidence exceeding 200 per 100,000 persons and a prevalence rete of 40 per 100,000 individuals) (Chen et al., 2024).

Isolated rib fractures are usually not fatal; however, they can lead to complications due to pain related hypoventilation and alterations in breathing mechanisms, which can be the cause of increased mortality especially in the frail or elderly population. Despite the frequency of chest injuries, there is no consensus on how to best manage pain related to chest wall trauma (Dogrul et al., 2020). Kinesiotaping (KT) is believed to play a role in reducing pain in musculoskeletal injuries, as it expedites recovery and decreases the likelihood of chronic pain development (Brockmann & Klein, 2018; Tang et al., 2025; Jiao, Tao & Cui, 2025; Kandeel et al., 2025; Bresgen et al., 2025). KT improves muscle function, blood and lymph circulation, reduces pain and supports joint function by reducing muscle tension and improving fascia and muscle function. KT increases stimulation of cutaneous mechanoreceptors and thereby improves proprioception (Jiao, Tao & Cui, 2025; Kandeel et al., 2025; Aksungur et al., 2025; Ghai, Ghai & Narciss, 2024; Kumbrink, 2014; Aldalati et al., 2025). Patients typically experience symptom relief, increased comfort, and greater stability in the affected area after KT application. KT also effectively reduces the sensation of pain after procedures involving chest injuries (Imperatori et al., 2016). A prominent symptom of rib fractures is intense pain worsened by chest movement, further compromising breathing mechanics. The most commonly affected ribs are the 5th to 9th (Kase, Wallis & Kase, 2003). Injury results in reduced respiratory volumes and coughing strength, leading to atelectasis, decreased lung compliance, and hypoxemia (Ho, Karmakar & Critchley, 2011). Poorly managed pain prolongs patient recovery and increases the risk of chronic pain development (Dunwoody et al., 2008). Treatment involves analgesics and rest, KT can be used as supportive therapy to provide support to injured muscles, bones, and joints, improve fascial function and position, and enhance segmental stability of the chest, facilitating the natural healing process. Simultaneously, by lifting the skin, KT accelerates blood circulation and lymphatic drainage, reducing pain due to decreased nociceptive stimulation (Kumbrink, 2014; Bakker et al., 2022). Immediately after the injury, in the first 24–72 h when swelling and hematoma are prominent, it would be appropriate to apply KT with lymphatic correction techniques, reducing inflammation and pain over the fracture or impact site. However, this technique does not provide sufficient support for fractured ribs, so often, immediately after chest injury with rib fractures, a muscle correction technique is preferred, reserving lymphatic correction for when swelling subsides (Kumbrink, 2014).

After thoracotomy or video-assisted thoracoscopic surgery (VATS), KT effectively reduced pain. According to existing studies, KT does not impact respiratory muscle strength; its primary effect is pain reduction (Imperatori et al., 2016). A study by Dal et al. (2024) analysed a small inhomogeneous sample of patients with rib fractures and found an isolated reduction of pain measured by VAS with KT treatment on day 4 only. It has already been called for more meticulously structured studies in the field of kinesiotaping in rib fracture (Tekin, 2024).

The purpose of our randomized controlled study was to determine the influence of KT on pain intensity and functional breathing parameters in patients with unilateral rib fracture upon enrolment and on the first day after inclusion in the study. Additionally, the aim was to assess the functional status of patients ten days after study inclusion. We hypothesised that KT reduces pain and improves respiratory function.

## Materials & Methods

The study was approved by the Slovenian National Medical Ethics Committee (No. 0120-110/2020/3). It was registered with ClinicalTrials.gov, “Influence of Kinesiotaping on Pulmonary Function Improvement in Multiple Rib Fracture”, identifier: NCT05753982.

The study was conducted in accordance with the principles of the Helsinki-Tokyo Declaration and the Code of Ethics for Healthcare Workers in Slovenia (World Medical Association, 2024).

A prospective, controlled study was conducted at the Clinical Department of Anaesthesiology and Surgical Intensive Therapy of the University Medical Centre Ljubljana by the Perioperative Respiratory Medicine Service, recruiting inpatient patients only.

Patients provided written informed consent to participate in the research.

Patients were randomly assigned to two groups, using a computer-generated list of random numbers: the study group (SG) and the control group (CG). Sealed envelopes with marked protocols were used sequentially. Both groups received standard respiratory physiotherapy (breathing exercises), while in the SG kinesiology tapes were applied. We used “Cure Tape^®^” (Netherlands, Thysol company), elastic, adhesive tape without medicines, made of 95% cotton, 5% spandex with an acrylic adhesive layer. The colour used was beige. KT was consistently applied by two expert physiotherapists, trained in NITA (Nasara International Taping Academy). Patients were monitored for signs of harm or adverse events (skin irritation, allergic reaction to taping glue, and similar).

Measurements of respiratory mechanics, saturation, expired carbon dioxide and pain levels were recorded and compared in both groups upon inclusion, after KT placement and respiratory physiotherapy (or respiratory physiotherapy only in the CG) and the following day and the EuroQol Group’s Health Questionnaire was applied remotely 10 days after inclusion (see below).

### Inclusion criteria

Adults (18 to 65 years old), isolated unilateral rib fracture (less than 5 ribs), willingness to participate in the research, and signing of the consent form.

### Exclusion criteria

Minors, pregnant women, contraindications for KT use (atrophic skin, patient non-cooperation, fresh wounds), patients incapable of active participation due to poor understanding, contraindications for spirometry, concomitant injuries and pneumothorax and/or haemothorax, penetrant chest injuries, operative rib fixation, regional anaesthesia for pain management. We also excluded patients with chronic analgesic opioid treatment.

### Patient inclusion

The research was conducted by two anaesthesiologists responsible for enrolling patients in the study, and physiotherapists assigned to the Traumatology Department on the day of the research, who performed the measurements. The process was not blinded because of the obvious presence or absence of kinesiology tapes.

### Clinical management

In both groups, measurements of respiratory mechanics and pain levels were recorded. Each set of respiratory measurements included: Spirometry using a portable spirometer (Vitalograph^®^ 6300 Micro, Vitalograph, Ennis, Ireland) in a semi-seated position, which recorded Forced Vital Capacity (FVC), Forced Expired Volume in the 1st second (FEV1) and Peak Expiratory Flow (PEF). Chest expansion was measured as the difference in chest circumference between inhalation and exhalation using a measuring tape. We also measured arterial oxygen saturation with a pulse oximeter and carbon dioxide levels in exhaled air (Masimo Kit Rad-97™ Pulse CO-Oximeter; Masimo, Irvine, CA, USA). Pain levels were objectively assessed with an algometer and subjectively with VAS scale (0 indicating no pain and 10 indicating the worst possible pain). Maximal inspiratory pressure (MIP) and maximal expiratory pressure (MEP) were also measured during an inspiration and expiration attempt with a blocked airway and expressed as obtained pressure in accordance with the American Thoracic Society (ATS) and the European Respiratory Society (ERS) statement (American Thoracic Society/European Respiratory Society, 2002). The first set of measurements was taken upon admission, after inclusion in the study. KT was then applied in the SG. Prior to application of KT skin was cleaned with soap and water and dried. Patients received tape application with 50% of tension in the middle third of the tape. Prepared tape for application was 5 cm wide and 20 cm long. Four tapes were applied—above, below, left and right from the injured area on the chest wall (Fig. 1). Following this, respiratory exercises (abdominal-diaphragmatic breathing exercises) were performed. Fifteen minutes after performing respiratory exercises (or applying kinesiology tapes and then performing breathing exercises in the SG group), the second set of measurements was conducted. We allowed a 30 min gap between KT application and the second set of measurements for KT to elicit an effect (see Fig. 2). The third set of measurements was taken the day after admission. On the day of inclusion in the study and the following day, analgesic medication consumption was recorded. Patients received paracetamol and atoricoxib at regular intervals and opioids (tapentadol) per demand. For the standardization of opioid doses, we compared the potency of tapentadol with morphine using the morphine milligram equivalent (MME) equation. In the calculation, we used a conversion factor of 0.4 for tapentadol.

**Figure 1:**
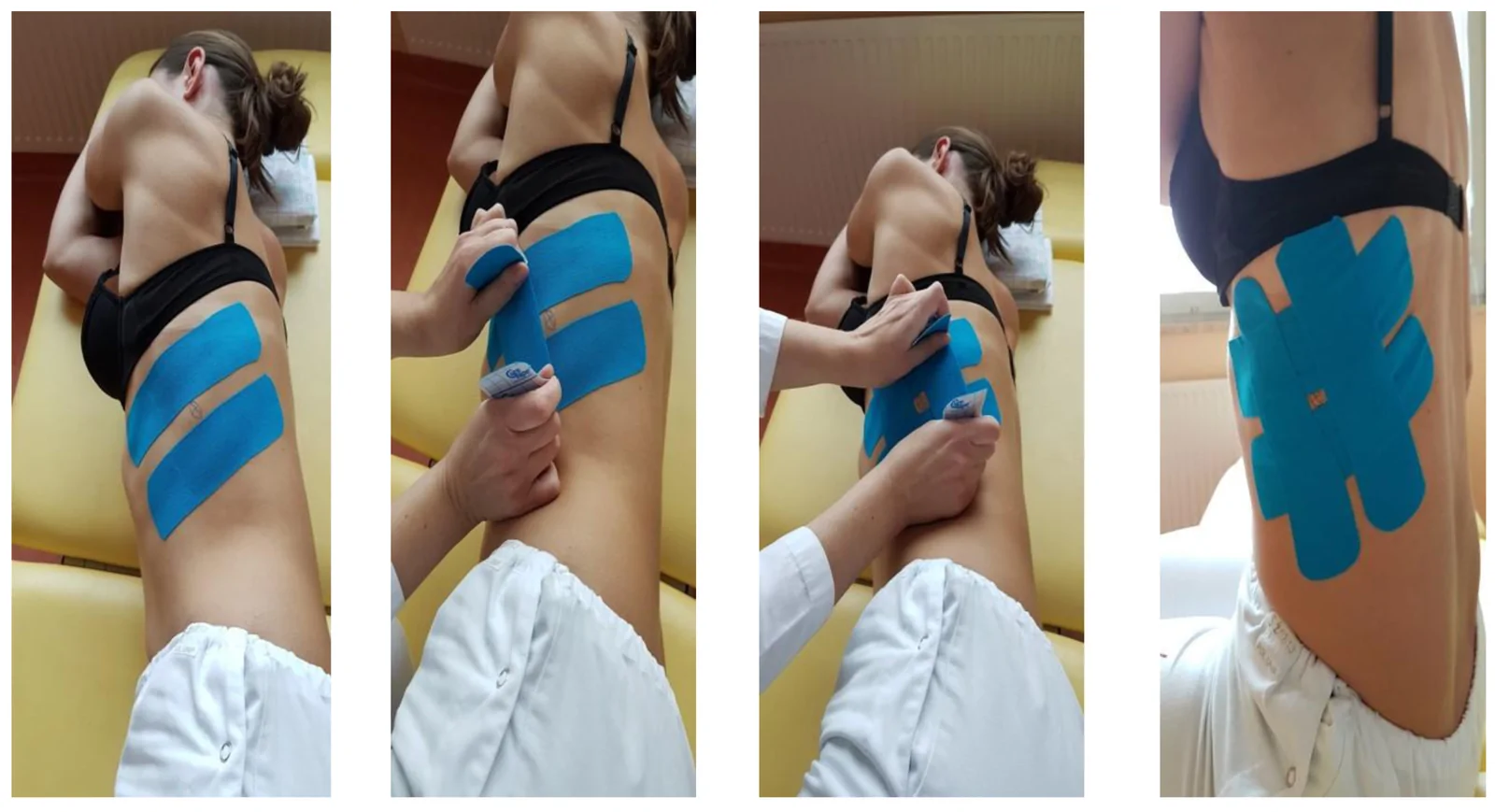
Kinesiotape application. Note: The subject in the images is a healthy volunteer. Blue tape was used as it is more visible in the images than beige, which was applied to the patients.

**Figure 2:**
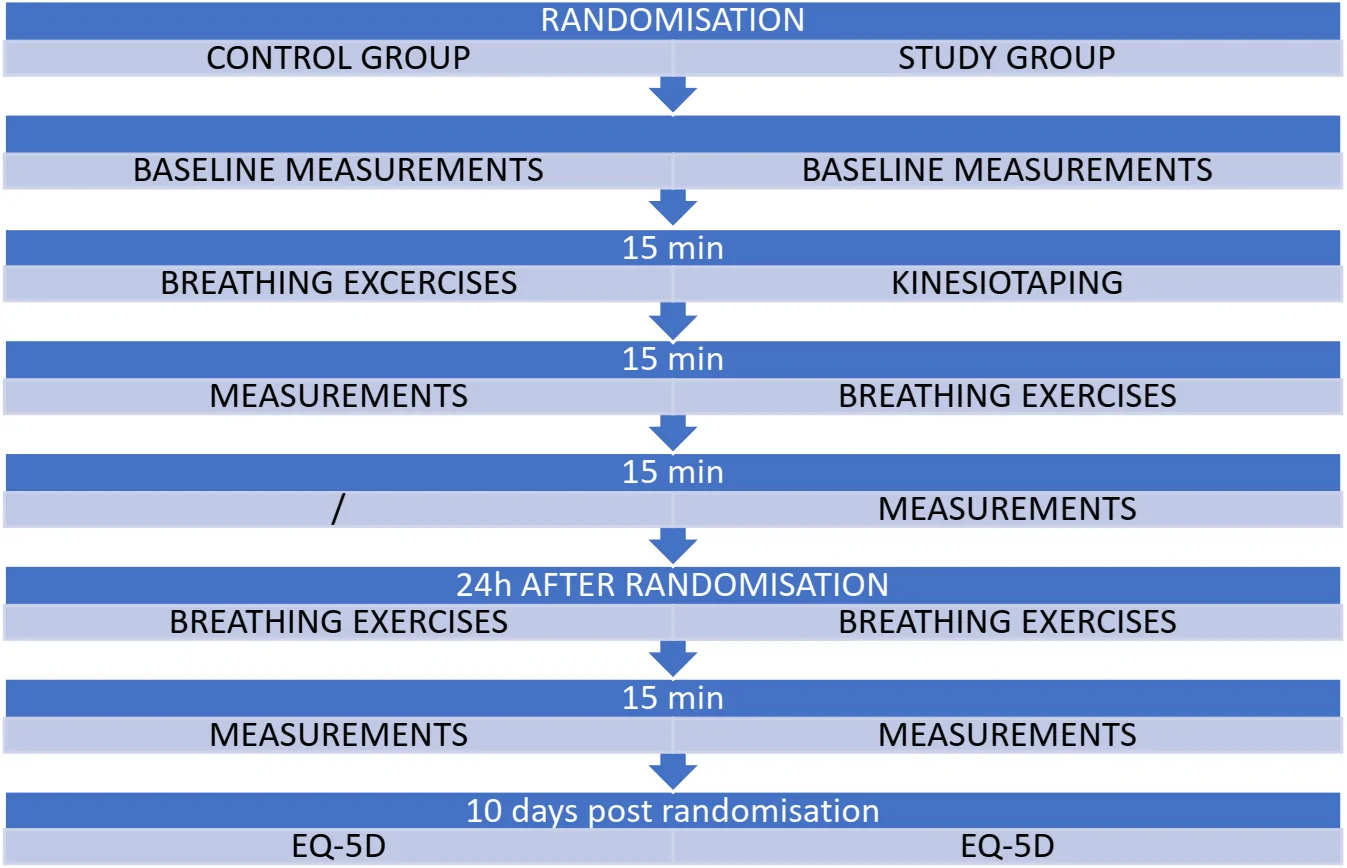
Clinical management flowchart. EQ-5D: EuroQol group’s health questionnaire.

MME = Tapentadol (mg) × 0.4

Ten days after inclusion, patients were contacted by phone for follow up questions, including avoiding possible harm, and they completed the EQ-5D (Fig. 2).

The EuroQol Group’s Health Questionnaire (EQ-5D) is a standardized questionnaire for assessing health status developed by the international multidisciplinary EuroQol Group. The questionnaire evaluates mobility, self-care, usual activities, pain/discomfort, and anxiety/depression. Each domain is scored from 1 to 5 points (zero, slight, moderate, severe, and extreme problems), where 1 indicates no problem and 5 indicates the most severe problem. EQ VAS records self-rated health on a vertical analogue scale from 0 to 100, where 100 represents the best and 0 the worst health the patient can imagine (EuroQol Group, 1990).

### Data collection

We monitored the following values: FVC, FEV1, PEF, chest expansion, arterial oxygen saturation and carbon dioxide levels in exhaled air, MIP/MEP, VAS pain assessment, algometer measurements, analgesic medication consumption, patient functionality (based on the EQ-5D score).

Primary outcome was improvement in pain scores, with secondary outcomes being reduction in analgesic medication consumption, improvement of respiratory parameters and improvement in patient functionality.

### Statistical methods

Baseline demographic characteristics between groups were compared using the *χ*^2^ test for categorical variables and the Mann–Whitney *U* test for continuous variables, as appropriate.

Respiratory variables and pain measurements are presented as median and interquartile range (IQR). Between-group comparisons at individual timepoints were performed using the Mann–Whitney *U* test. Within-group changes over time were assessed using the Wilcoxon signed-rank test.

Associations between continuous variables, including spirometry parameters, pain intensity (VAS), morphine consumption, and age, were evaluated using Spearman’s rank correlation coefficient.

The relationship between morphine consumption and anxiety levels over time was analysed using a nonparametric repeated-measures approach. Due to the exploratory nature of the study and the non-normal distribution of the data, results are presented descriptively, supported by rank-based correlation analyses rather than parametric mixed-effects modelling.

Relative changes in algometry measurements were calculated and compared between groups using the Mann–Whitney *U* test.

Questionnaire results (EQ-5D) are presented as median (IQR) and compared between groups using the Mann–Whitney *U* test.

Histograms were constructed to visualize the distribution of morphine reduction rates in each group, allowing for qualitative assessment of variability and distribution patterns.

This study was designed as an exploratory pilot randomized trial to assess feasibility and estimate variability of key outcomes for future studies. Accordingly, no a priori sample size calculation was performed. Emphasis is placed on effect size estimation and distributional patterns, while *p*-values are interpreted cautiously.

All analyses were performed using R (R Core Team, 2021), R Foundation for Statistical Computing, Vienna, Austria. Statistical significance was defined as *p* < 0.05. Given the exploratory nature of the study, no correction for multiple comparisons was applied.

## Results

Forty-five consecutively admitted trauma patients with unilateral isolated chest injury (fracture <5 ribs) were identified for inclusion in the study in the Traumatology Department. Patients were assessed for eligibility to participate in the study. Seven did not meet inclusion criteria, four patients refused to participate. Others were randomly assigned to two groups in accordance with standard randomization procedure: the study group (SG) consisting of 19 patients and the control group (CG) consisting of 15 patients. After enrolment, two additional patients in the SG declined further participation. Ten days after inclusion, five patients (two in SG and three in CG) did not respond to a phone call to complete the EuroQol Group’s Health Questionnaire (EQ-5D) (Fig. 3).

Missing data were handled according to a modified intention-to-treat approach, including all randomized participants who had at least one post-baseline measurement. Two patients in the study group withdrew immediately after randomization and were excluded from the efficacy analysis but remained accounted for in the participant flow diagram. For the EQ-5D questionnaire, five patients were lost to follow-up and therefore not included in that analysis.

No harmful events were recorded.

Demographic data of included patients is shown in Table 1, respiratory variables in Table 2.

**Figure 3:**
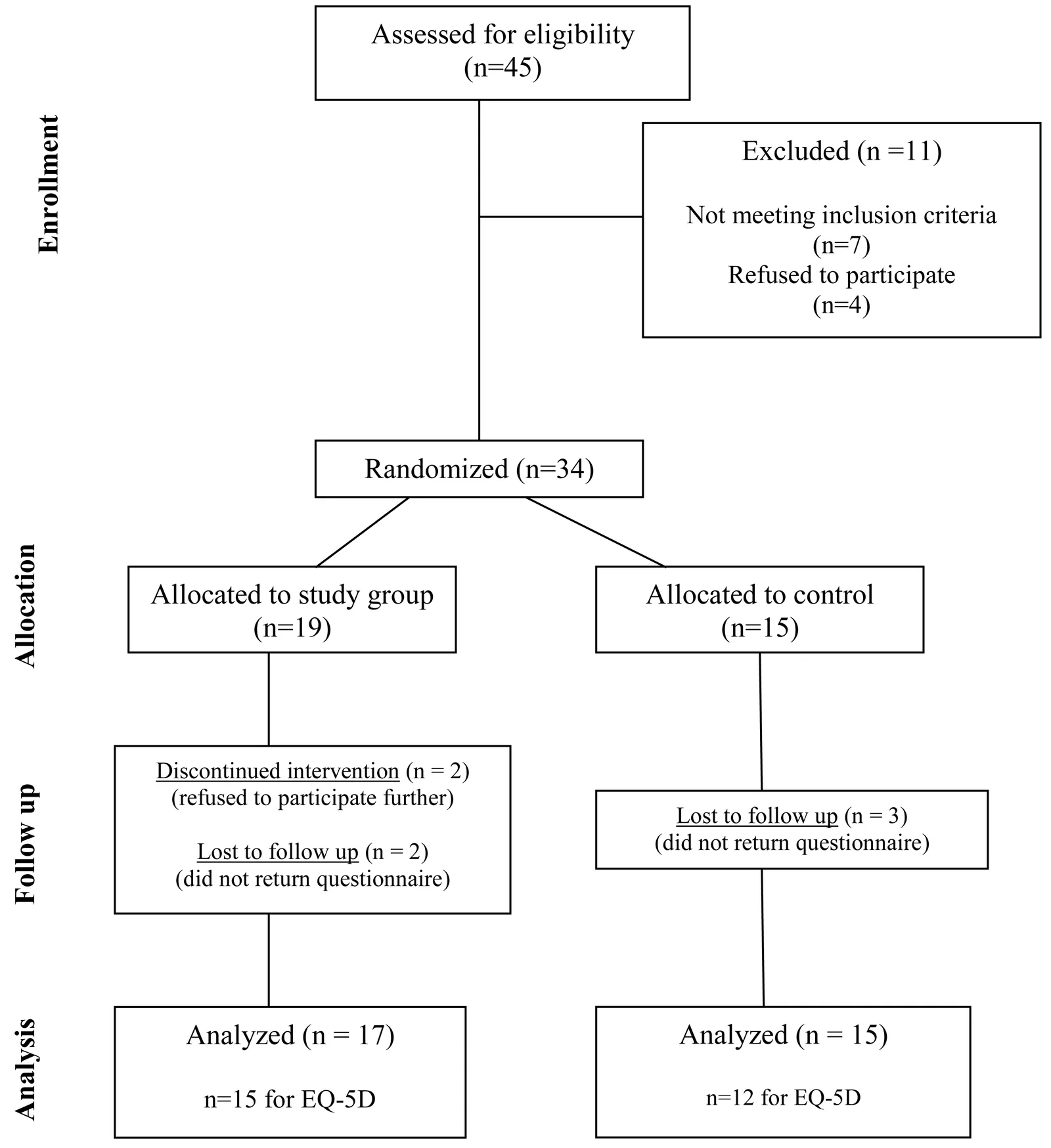
Patient inclusion and exclusion flowchart.

Respiratory and pain parameters at enrolment (0-a), after the first session of respiratory physiotherapy with or without kinesiotaping (0-b), and on the following day (1) are presented in Table 2. Continuous variables are expressed as median and interquartile range (IQR). Between-group comparisons at each timepoint were performed using the Mann–Whitney *U* test.

Additionally, between-group differences in change over time (0-a to 0-b, 0-a to 1, and 0-b to 1) were assessed using the Mann–Whitney *U* test. No statistically significant differences between the study and control groups were observed for any of the evaluated variables, including VAS, FVC, FEV1, PEF, thoracic excursion, MEP, SpO_2_, EtCO_2_, and algometry (all *p* > 0.05). A borderline difference was observed for MEP change between 0-a and 0-b (*U* = 181, *p* = 0.05), but this was not sustained in subsequent comparisons.

Pain characteristics in both groups are compared in Table 3, correlation between pain and morphine consumption is shown in Fig. 4, correlation between age and pain evaluation in Fig. 5, relative change in algometry after placement of KT in Fig. 6 and morphine reduction rates in Fig. 7.

The histogram compares the distribution of morphine reduction rates between the two groups, allowing us to see the frequency and range of morphine reduction. In the study group the distribution is more spread out, with a higher frequency of significant reductions in morphine use. In the control group the distribution is narrower and centred around lower reduction rates. This implies that morphine uses in the control group tended to decrease less drastically, with fewer patients experiencing large reductions in morphine consumption.

Morphine consumption on the intervention day has a weak positive correlation with anxiety levels (Spearman’s *ρ* = 0.175).

**Table 1:** Demographic data.

**Gender**	**Study**	**Control group**	**Statistical test**
*Male*	*11*	*10*	*Fisher test,p* = 0*.66*
*Female*	*4*	*2*	
Age (years), median [IQR]	48.00 [43.50, 53.00]	59.00 [48.50, 63.00]	(Mann–Whitney), *p* = 0.051

**Table 2:** Respiratory variables.

Variable (timepoint)	Control group (CG), median [IQR]	Study group (SG), median [IQR]	Comparison	Mann– Whitney *U*	*p*-value
VAS, 0-a	6.00 [3.50, 7.00]	6.00 [3.00, 7.00]	0-a to 0-b	125	0.95
VAS, 0-b	5.00 [3.50, 6.00]	5.00 [2.00, 7.00]	0-a to 1	132	0.86
VAS, 1	4.00 [2.25, 6.20]	4.00 [2.50, 6.00]	0-b to 1	140	0.64
FVC (ml), 0-a	2,990 [2,065, 3,675]	2,920 [2,350, 3,990]	0-a to 0-b	140	0.64
FVC (ml), 0-b	3,010 [2,010, 3,310]	3,030 [2,460, 4,310]	0-a to 1	156	0.28
FVC (ml), 1	3,200 [2,100, 3,480]	3,090 [2,380, 4,380]	0-b to 1	147	0.46
FEV1 (ml), 0-a	2,260 [1,545, 2,885]	2,460 [1,850, 3,000]	0-a to 0-b	146	0.48
FEV1 (ml), 0-b	2,270 [1,445, 2,595]	2,520 [2,000, 2,910]	0-a to 1	161	0.21
FEV1 (ml), 1	2,220 [1,530, 2,785]	2,490 [1,720, 3,280]	0-b to 1	143	0.57
PEF (L/min), 0-a	280 [194, 377]	369 [226, 437]	0-a to 0-b	160	0.23
PEF (L/min), 0-b	287 [183, 351]	367 [240, 434]	0-a to 1	165	0.16
PEF (L/min), 1	281 [200, 393]	380 [319, 413]	0-b to 1	151	0.39
Circumference difference, 0-a	0.60 [0.40, 1.75]	1.00 [0.40, 1.50]	0-a to 0-b	137	0.72
Circumference difference, 0-b	1.00 [0.50, 1.25]	1.00 [0.40, 2.00]	0-a to 1	138	0.70
Circumference difference, 1	1.00 [0.60, 1.75]	1.50 [0.50, 3.00]	0-b to 1	145	0.51
MEP, 0-a	68.00 [51.50, 109.50]	85.40 [64.00, 112.00]	0-a to 0-b	181	0.05
MEP, 0-b	67.00 [52.60, 94.75]	75.00 [65.50, 91.20]	0-a to 1	150	0.41
MEP, 1	55.00 [47.10, 105.00]	101.00 [75.20, 120.00]	0-b to 1	158	0.25
SpO2, 0-a	96.00 [95.00, 96.50]	96.00 [95.00, 97.00]	0-a to 0-b	159	0.23
SpO2, 0-b	96.00 [95.00, 96.50]	96.00 [95.00, 97.00]	0-a to 1	138	0.68
SpO2, 1	96.00 [94.00, 97.00]	96.00 [95.00, 97.00]	0-b to 1	140	0.63
EtCO2, 0-a	4.00 [3.30, 4.45]	4.40 [4.00, 4.85]	0-a to 0-b	156	0.28
EtCO2, 0-b	4.40 [3.70, 4.60]	4.40 [4.00, 4.70]	0-a to 1	165	0.16
EtCO2, 1	4.20 [3.50, 4.65]	4.60 [4.00, 4.80]	0-b to 1	144	0.53
Algometry, 0-a	2.10 [1.62, 3.48]	2.10 [1.50, 3.50]	0-a to 0-b	124	0.92
Algometry, 0-b	2.20 [1.58, 3.25]	2.75 [1.26, 4.25]	0-a to 1	143	0.56
Algometry, 1	2.70 [1.65, 3.67]	2.80 [1.60, 5.10]	0-b to 1	140	0.65

Morphine consumption on the second day shows almost no correlation with anxiety levels (Spearman’s *ρ* = 0.008).

Patients in the study group experienced a reduction in pain as measured with VAS from baseline (0-a) to post-intervention (1). The median decrease in pain intensity was approximately 1 point on the 0–10 scale; however, this change did not reach statistical significance when analysed using the Wilcoxon signed-rank test (*p* > 0.05). Despite the lack of statistical significance, individual patients demonstrated clinically relevant improvements, for example a reduction from VAS 6.0 to 4.0.

**Table 3:** Pain characteristics.

**Pain indicator**	Study group (median, IQR)	Control group (median, IQR)	*p* value **(Mann–Whitney U test)**
Pain (VAS) before intervention	6.0 (3.5–7.0)	6.0 (4.0–7.0)	0.917
Pain (VAS) after intervention	5.0 (2.5–7.0)	5.0 (4.5–6.0)	0.979
Pain (VAS) the day after intervention	4.0 (2.8–6.0)	5.0 (3.0–6.2)	0.896
Algometer scale before intervention (kg/cm^2^)	1.8 (1.5–3.4)	3.0 (1.6–4.0)	0.451
Algometer scale after intervention (kg/cm^2^)	2.5 (1.1–3.8)	2.8 (1.9–3.6)	0.736
Algometer scale second day (kg/cm^2^)	2.3 (1.5–4.9)	3.3 (2.4–4.0)	0.550
Morphine consumption day 0 (mg)	40 (30–60)	20 (20–60)	0.449
Morphine consumption day 1 (mg)	20 (20–40)	20 (10–30)	0.289

**Figure 4:**
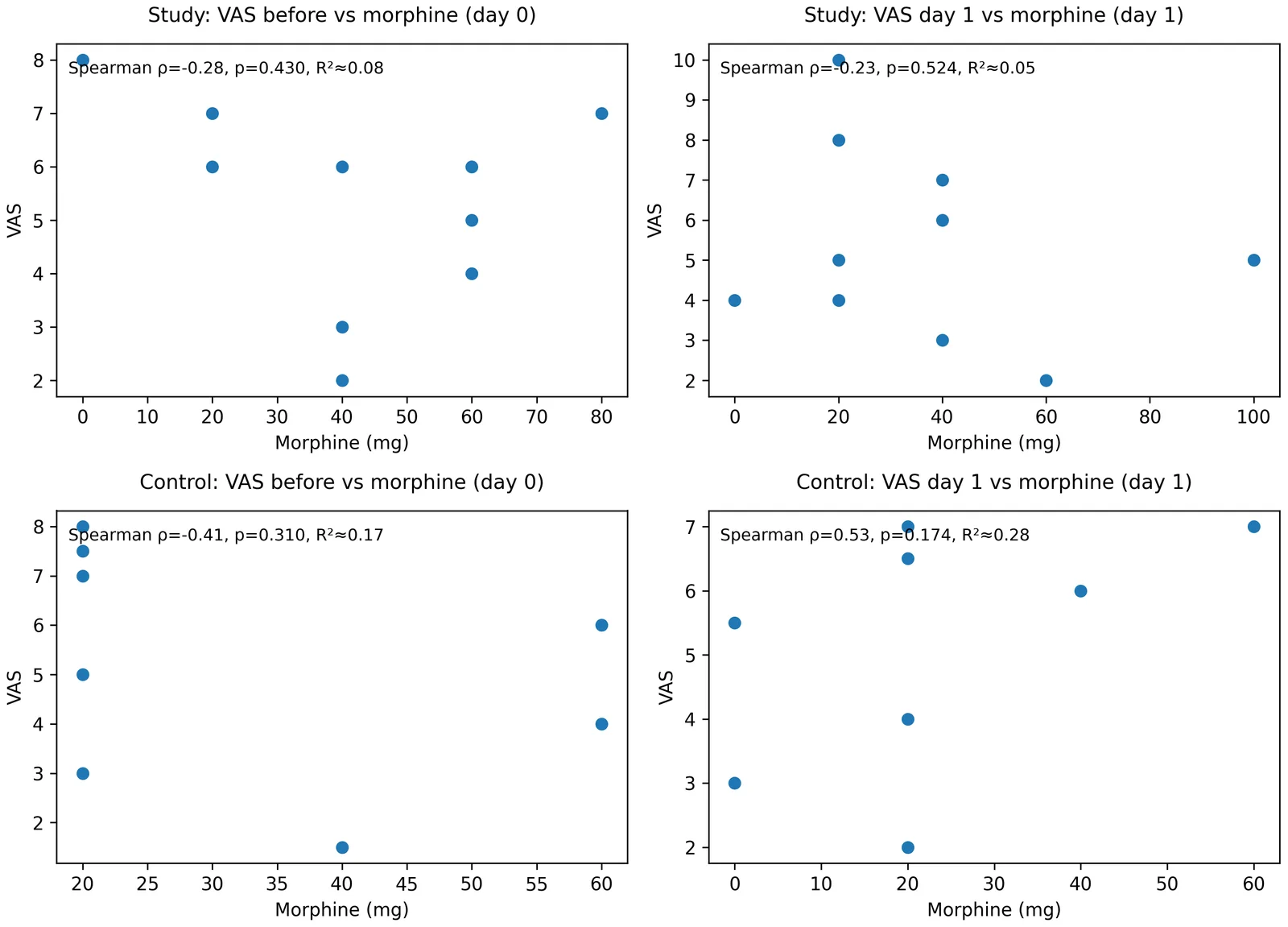
Correlation between pain and opioid consumption.

**Figure 5:**
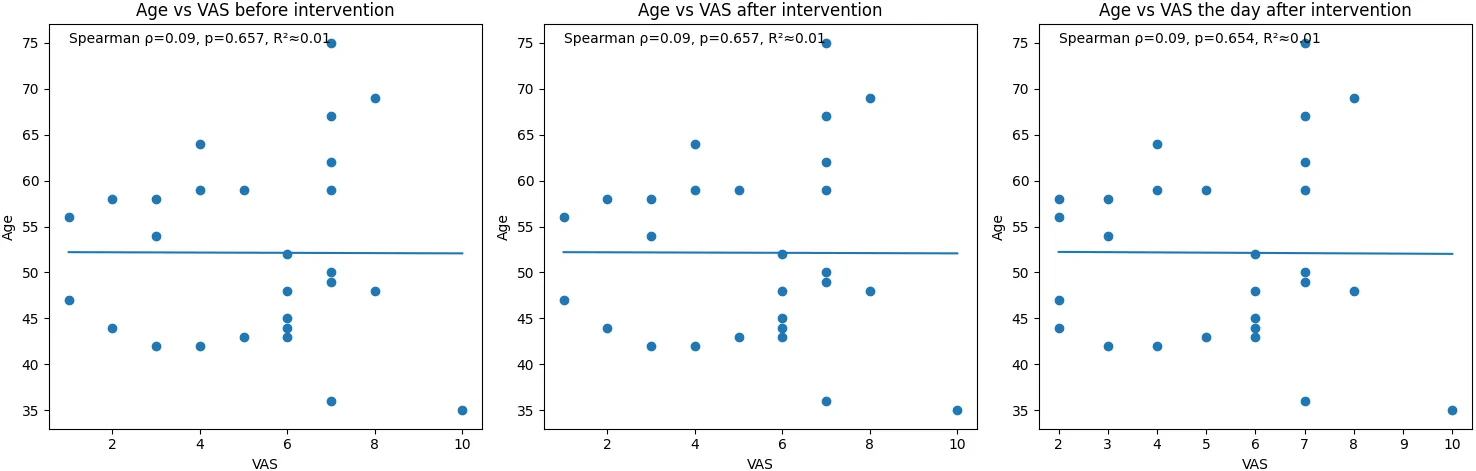
Correlation between age and pain.

**Figure 6:**
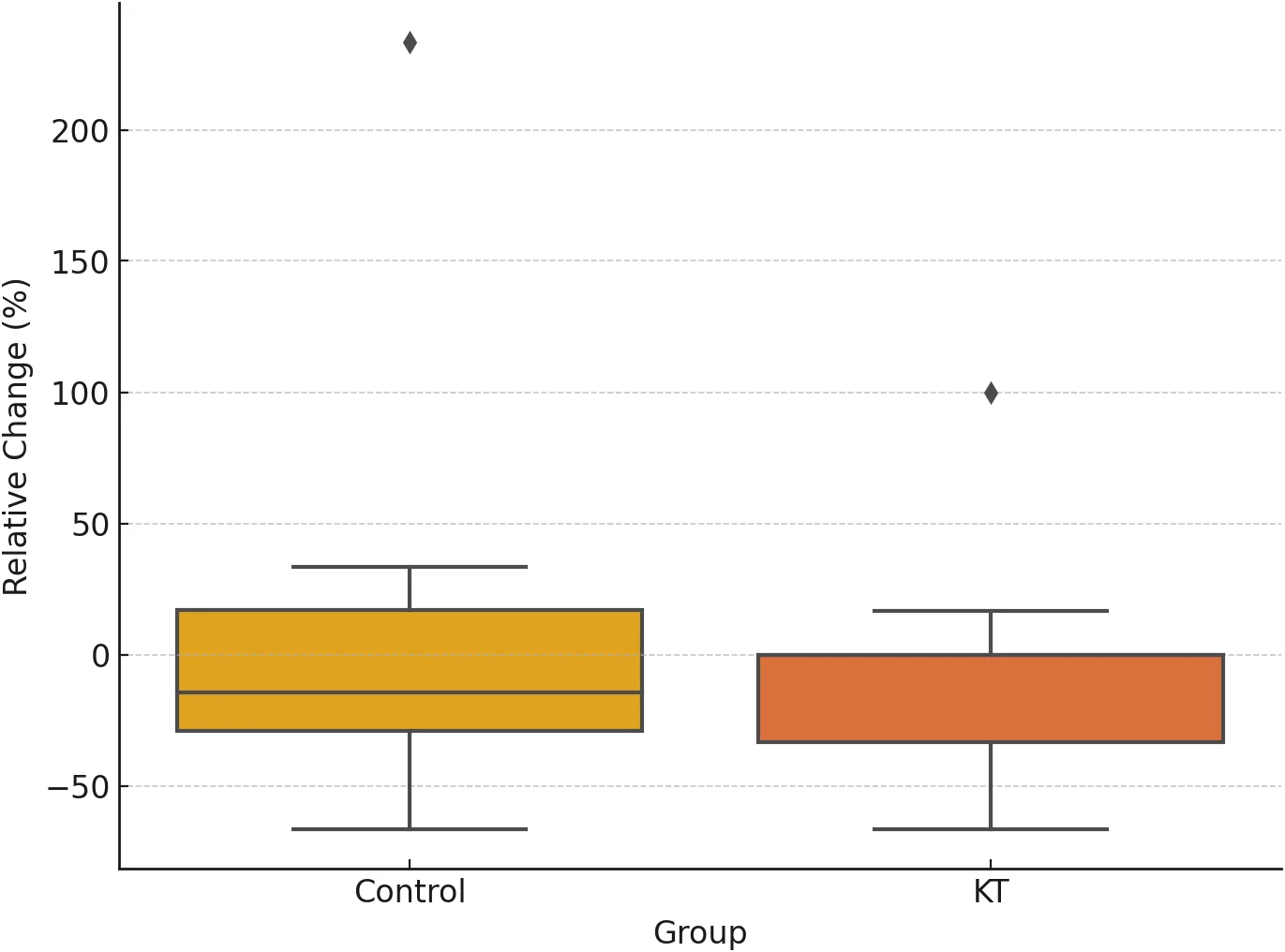
Relative change in algometry after placement of KT.

**Figure 7:**
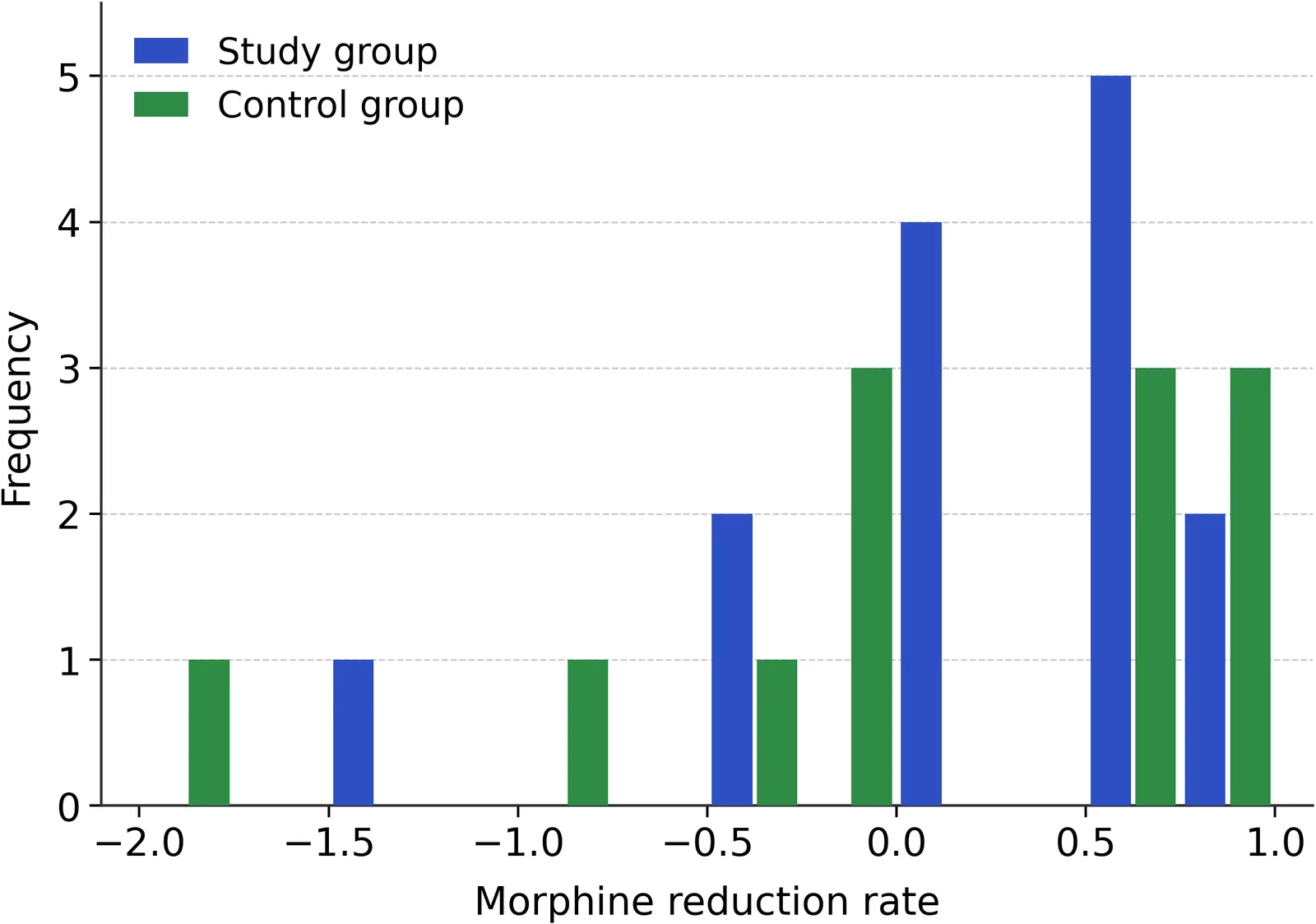
Morphine reduction rates.

No statistically significant change in pain levels was observed in the control group (Wilcoxon signed-rank test: median change −1.0, *W* = 15.0, *p* = 0.11). The response pattern in this group was heterogeneous, with some patients showing improvement (*e.g.*, ΔVAS = −2.0), while others experienced worsening of pain (*e.g.*, ΔVAS = +3.0).

### Analysis of variance of VAS scores

Dispersion of VAS scores over time was assessed descriptively using interquartile ranges (IQR) and formally compared using a nonparametric test for equality of variances (Fligner–Killeen test).

In the control group, variability in VAS scores showed a slight decrease from baseline (0-a) to the day after intervention (1), indicating a trend toward a more uniform response over time. In contrast, the study group demonstrated an increase in variability, reflected by wider interquartile ranges at follow-up, suggesting a more heterogeneous response to the intervention (Fig. 8).

**Figure 8:**
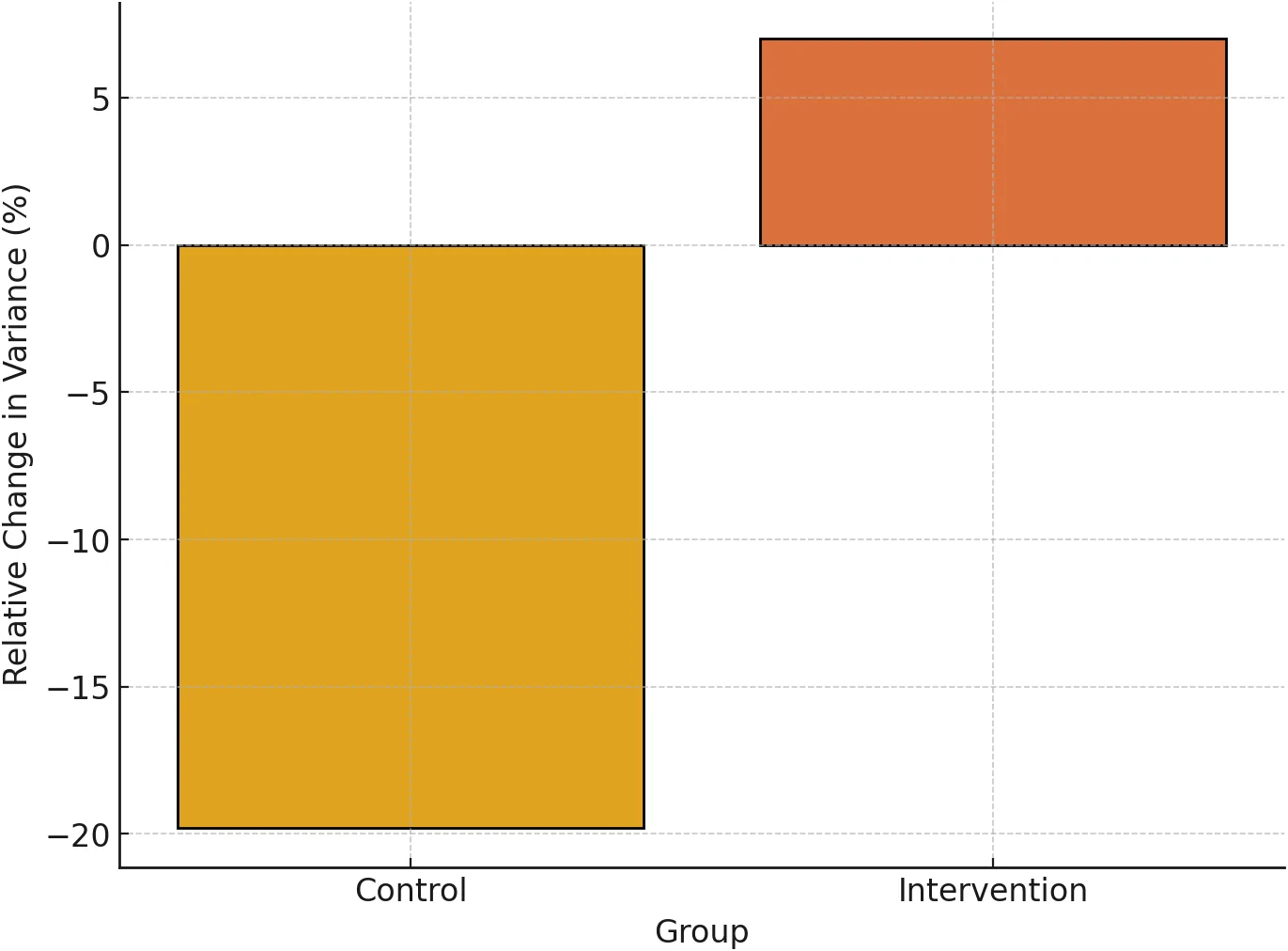
Variability in VAS scores.

Formal comparison of dispersion did not demonstrate a statistically significant difference in variability over time in either group (Fligner–Killeen test: control group *p* = 0.750; study group *p* = 0.863).

These findings indicate that, although the study group visually suggests the emergence of distinct response patterns—consistent with the presence of both good and poor responders—this heterogeneity was not confirmed.

Questionnaire results are presented in Table 4.

## Discussion

In this pilot randomized controlled trial, KT did not improve respiratory function, nor did it reduce pain in unilateral rib fracture. Rib fracture is associated with several possible complications (Anderson et al., 2025). Airway clearance can be reduced due to altered respiratory mechanics and pain and this in turn can lead to pulmonary complications (Belli et al., 2021). KT could in theory address both issues, however, this pilot study did not yield results that would support the use of KT for this purpose.

### Impact of KT on respiratory mechanics in chest injuries

There are no studies in the literature that specifically examine the impact of KT on respiratory mechanics in chest injuries. Previous research explored the effect of KT on respiratory muscle support in patients with COPD. KT improved all measured respiratory parameters in COPD patients, except FVC (Metin Ökmen et al., 2020). Studies suggest that KT enhances lung function and functional capacity in COPD patients (Tomruk et al., 2020; Ganesh, Praneetha & Naik, 2023; Alsaeedi et al., 2021). Shah & Borkar (2024) report positive results of KT on pulmonary function in children with pneumonia. There is also literature with negative results, for example a review article by De Campos, Neves & Isoppo (2023), who found that KT did not improve lung function in COPD patients. One more study of KT in COPD is currently being run (Zeng, Tian & Xiao, 2021). It is important to note that the altered respiratory mechanics observed in chest injuries and pneumonia is different from that seen in COPD patients. Chest injuries are characterized by intense pain, aggravated by movement of the affected body part, leading to a reduction in the absolute chest circumference during breathing, reduced amplitude of chest circumference fluctuation, decreased respiratory capacity, and weakened strength of cough. These factors can contribute to the development of atelectasis, reduced lung compliance, hypoxemia, and respiratory distress (Ho, Karmakar & Critchley, 2011).

**Table 4:** EQ-5D questionnaire results.

**Activity**	**Study group median (IQR)**	**Control group median (IQR)**	*p*-value (Mann–Whitney U test)
Walking	2.00 [1.00, 2.00]	1.00 [1.00, 2.25]	0.216
Hygiene	3.00 [2.00, 3.00]	2.00 [1.00, 2.00]	0.086
Activity	3.00 [2.00, 4.00]	3.00 [2.75, 4.00]	0.860
Pain	3.00 [2.00, 3.50]	3.00 [2.00, 3.25]	0.857
Anxiousness	1.00 [1.00, 2.00]	1.00 [1.00, 1.25]	0.380

In our study, there were no differences between SG and CG in respiratory parameters and chest wall expansion, with the exception of maximal expiratory pressure (MEP) being significantly higher in the control group after the first set of respiratory physiotherapy on the day of inclusion. This result was not sustained the following day.

### Impact of KT on pain in chest injuries

The impact of KT on reducing pain in chest injuries has previously been investigated in patients with uncomplicated isolated rib fractures Bakker et al. (2022). demonstrated that application of KT reduced pain resulting from chest injuries. Similar findings of reduced pain in chest injuries with KT application have been reported by other researchers (Dafoe, Rushworth & Stiller, 2020; Akça, Şaşmaz & Kaplan, 2020; Sareen, Jain & Pagare, 2015). Even after procedures involving rib osteotomy, such as lobectomy for lung cancer or sternotomy, KT application has been associated with decreased pain (Brockmann & Klein, 2018; Imperatori et al., 2016). In our exploratory randomized study, KT placement was not associated with a reduction in pain between SG and CG. We also did not find clear differences in opioid consumption between the groups. However, a trend towards reduced opioid consumption was observed within both groups.

Substantial reductions in morphine consumption between the intervention day and the second day were more frequent in the study group. Overall, the study group appears to have more pronounced reductions in morphine use compared to the control group, which could indicate the intervention’s effectiveness in reducing pain or reliance on morphine.

By analysing the variance of VAS scores, we showed an increased variance in the SG, suggesting that not all patients benefited equally. The possibility of good responders and poor responders to KT is an interesting concept that warrants investigation in further studies.

The non-significant results may be attributed to the small number of included patients in this pilot study and a stringent design of inclusion and exclusion criteria, which produced a more uniform group of investigated individuals. Pain perception may also have been influenced by the study protocol, where respiratory physiotherapy was performed immediately before pain measurements, potentially exacerbating pain. Despite limiting eligible age to 65 years, our control group was almost significantly older than the study group, an unfortunate result of the strict randomization process. Pain thresholds increase with age, which could explain a non-significant trend towards better respiratory mechanics measurements in the older (control) group (Lautenbacher et al., 2017). It should also be mentioned that morphine consumption was weakly correlated with anxiety, a fact that could have confabulated the results.

Despite a considerable number of chest injury patients that are admitted to our hospital, only a few had isolated unilateral rib fractures (less than 5 ribs). In a study investigating the impact of KT on isolated rib injuries in the emergency department, Akça and colleagues included 82 patients with rib injuries, but only 30 patients met strict criteria. Nevertheless, they managed to show that KT combined with nonsteroidal anti-inflammatory drugs more effectively reduced pain than treatment with analgesics alone (Akça, Şaşmaz & Kaplan, 2020). In other studies, examining the impact of KT on chest injuries, patient inclusion criteria were less stringent. For instance, Bakker et al. (2022) demonstrated pain reduction with KT placement after uncomplicated traumatic shoulder or chest injuries. Since such a patient group is heterogeneous, the reliability of the results is questionable. Pain in isolated rib injuries differs from that in combined injuries or isolated shoulder injuries. A small study by Dal et al. (2024) showed a reduction of pain in KT group on day four after inclusion, we could not confirm that as our patients were discharged sooner. However, their patient selection did not limit the number of injured ribs and it is uncertain if any of the patients had bilateral rib injury, again producing a heterogenous sample. None of these studies, tried to measure pain using an objective method like algometry. Our algometry measurements, however, did not yield any significant results.

Age was not a significant factor in pain score (Fig. 3). A meta-analysis by Lautenbacher et al. (2017) showed that increasing age increases pain threshold for low intensity pain, but has no effect on pain tolerance. A review by Eltumi & Tashani (2017), however, suggests increasing age can either increase or decrease pain threshold. The relationship between pain and age is also difficult to assess due to altered cognition and sensory impairment (Mullins et al., 2022). This could all explain our results: pain from rib fracture is not of low intensity (mean VAS reported before intervention was 6) and altered cognition might have interfered with reported VAS scores. Also, by study design, our patients were all between 18 and 65 years of age with the intention to eliminate the influence of advanced age to pain perception and reporting.

### Health questionnaire EQ-5D-5L

In our study, 10 days after the injury, there were no noticeable differences between the groups in the impact of the injury on walking, washing, dressing, pain, anxiety, and overall health assessment. Morphine consumption was independent of anxiety level. The lack of differences between the groups could be attributed to effective analgesic therapy, enabling better functioning and subjectively reduced perception of the injury’s impact on health status. On the other hand, after discharge from the hospital, no one reattached KT to patients when they came loose.

### Significance and limitations of the study

The conducted exploratory study is the first to compare the effect of KT on respiratory mechanics measurements in chest injuries with rib fractures. No similar studies were found in the literature. The strength of the study lies in its prospective and randomized design. Additionally, the study’s advantage is the comprehensive assessment of pain, including both subjective measurement with the VAS scale and objective measurement with an algometer. Other studies examining the impact of KT on pain use only subjective pain measurements with the VAS scale.

A limitation of the study is the small sample size, likely contributing to some parameters approaching statistical significance without reaching it. Many patients did not meet our strict inclusion criteria. Numerous patients were discharged to home care the day after the injury, making them ineligible for study inclusion. Another limiting factor is that due to the work process, two separate physiotherapists applied the intervention. They were, however, equally trained. The study was also not blinded due to obvious reasons of visible kinesiology tapes, presenting an additional limitation.

## Conclusions

The use of KT did not significantly improve respiratory mechanics, saturation or ventilation in this pilot randomized controlled study. There was no reduction of pain, neither evaluated with a subjective nor with the objective measurement to suggest a role of KT on pain reduction in unilateral isolated rib fractures. There is, however, evidence to suggest good and poor responders to KT. Further and larger studies of similar design are needed to further investigate the role of KT in isolated rib fractures.

## Supplemental Information

10.7717/peerj.21753/supp-1 Supplemental Information 1 Anonymized raw dataset

10.7717/peerj.21753/supp-2 Supplemental Information 2 CONSORT checklist

10.7717/peerj.21753/supp-3 Supplemental Information 3 Raw data codebook

10.7717/peerj.21753/supp-4 Supplemental Information 4 Trial protocol - in Slovenian

## Additional Information and Declarations

### Data Availability

The following information was supplied regarding data availability: The raw data is available in the Supplemental Files.

### Clinical Trial Registration

The following information was supplied regarding Clinical Trial registration: NCT05753982
