# Supplementary material for: The impact of kinesiotaping on functional respiratory outcome in chest injuries—a pilot randomized trial
Source: PeerJ. 2026 Oct 12;14:e21753. doi: 10.7717/peerj.21753 (PMC13671238; doi:10.7717/peerj.21753)
Supplement: Supplementary file 2 [file peerj-14-21753-s003.docx]

**The impact of elastic adhesive strip placement on functional respiratory outcome in chest injuries**

CODEBOOK

0-a (day 0, before intervention)

0-b (day 0, after intervention)

1 (day 1)

KT – kinesiotaping (study) group

M – male

F – female

VAS – visual analogue scale (1-10)

FVC – forced vital capacity (ml)

FEV1 – forced expiratory volume in first second (ml)

PEF – peak expiratory flow (L/min)

Circumference – chest circumference (m)

MIP - maximal inspiratory pressure (cmH2O)

MEP – maximal expiratory pressure (cmH2O)

Algometry (kg)

MORFINE – morphine (morphine milligram equivalents)

SpO2 – pulse oxymetry (%)

EtCO2 – exhaled tidal CO2 (kPa)

WALKING, HYGIENE, ACTIVITIES, PAIN, ANXIETY – parts of The EuroQol Group's Health Questionnaire (EQ-5D) (each part gives 1-5 points)

SCORE – score in EQ-5D
