# Supplementary material for: The impact of kinesiotaping on functional respiratory outcome in chest injuries—a pilot randomized trial
Source: PeerJ. 2026 Oct 12;14:e21753. doi: 10.7717/peerj.21753 (PMC13671238; doi:10.7717/peerj.21753)
Supplement: Supplementary file 3 [file peerj-14-21753-s004.docx]

**KINESIOTAPING- PROTOKOL**

Paciente bo v raziskavo vključil zdravnik iz tima respiratorne terapije, jim razložil namen in potek raziskave. Vsi pacienti, ki se bodo za sodelovanje odločili, bodo podpisali soglasje. Pri raziskavi bodo sodelovali zdravniki specialisti in specializanti anesteziologije in fiziatrije ter respiratorni fizioterapevti.

V raziskavo bomo vključili vse zaporedno sprejete kirurške paciente s poškodbo in/ali okvaro prsnega koša. Ob vključitvi zdravnik, presodi, če je pacient primeren za vključitev (vključujemo bolnike, ki imajo enostranske poškodba prsnega koša, ne vključujemo bolnikov z nestabilnim prsnim košem).

Izključitveni kriteriji bodo: mladoletnost, nosečnice, kontraindikacije za uporabo kinezio trakov (atrofična koža, nesodelovanje bolnika, sveža rana), bolniki, ki zaradi slabega razumevanja niso zmožni aktivno sodelovati pri raziskavi, kontraindikacije za spirometrijo.

Paciente bomo randomizirali v dve skupini: intervencijska skupina bo deležna terapije s kinezio trakovi (KT) (podporna tehnika namestitve trakov – ligamentna, po predlogi dr. Frangež), kontrolna skupina pa ne. Pri obeh skupinah se bo izvajala standardna terapija- trebušno/preponske dihalne vaje.

V vsako skupino bomo vključili 20 pacientov. Pri obeh skupinah bomo beležili meritve mehanike dihanja in stopnjo bolečine.

Posamezen sklop meritev bo zajemal: spirometrijo v polsedečem položaju (forsirana vitalna kapaciteta, FVC, forsiran ekspiratorni volumen v prvi sekundi, FEV1), največji pretok med izdihom- PEF, razliko v obsegu prsnega koša med vdihom in izdihom, mišično moč (MIP/MEP), stopnjo bolečine in podajnost tkiva. Za potrebe merjenja mišičnega tonusa bomo uporabili myoton, stopnjo bolečine bomo merili z algometrom in VAS, spirometrijo pa s (prenosnim) spirometrom.

Prvi sklop meritev bo narejen ob vključitvi v raziskavo, 2. sklop en dan po vključitvi v raziskavo (dogovor z lečečim zdravnikom, da se bolniki z oddelka ne odpustijo prej) in ev. 3. sklop meritev pri še vedno hospitaliziranih pacientih 5. dan od vključitve (zadnjo meritev se lahko izvede 5.-7. dan). Pri vseh meritvah bomo beležili vrsto in dnevno porabo protibolečinskih zdravil.

Ob odpustu bodo bolniki dobili za domov natančna navodila o dolžini namestitve trakov in tehniki odstranjevanja.

Kontrolni razgovor s pacientom bomo opravili 10. dan po telefonu (VAS, poraba analgetikov).

V mapi v IV. nadstropju bodo: protokol študije, list za vključevanje bolnikov (randomizacija-ovojnice), opisi ligamentne tehnike, srajčke za vsakega bolnika (notri bo soglasje, list za vpisovanje, navodila za bolnika za domov).

Če bodo vključeni bolniki ležali na stari travmatološki kliniki, so lahko med izvajanjem meritev papirji za bolnika tudi na tej lokaciji, vendar jih je potrebno po zaključku obravnave vrniti v mapo v IV. nadstropje.

**VKLJUČITEV BOLNIKA V ŠTUDIJO**

- prepoznava bolnika - fizioterapevt, zdravnik razpisan na respiratorni fizioterapiji; bolnika tudi vprašata (eden od njiju) o načelni privolitvi za sodelovanje v študiji
- obvestiti koordinatorja anesteziologa (tel. št. v mapi)
- koordinator poskrbi za randomizacijo, pojasnilo in pridobitev soglasja bolnika, obvesti fizioterapevta o pridobitvi soglasja, da ta lahko izvede meritve in KT, Majo Potočnik, ki skrbi za izvedbo meritev s strani fizioterapevtov pri vseh sklopih.
- ob podpis soglasja je potrebno zapisati telefonsko številko bolnika,
- dogovor z lečečim zdravnikom, da bolnik ostane hospitaliziran vsaj 2 dni
- po izvedenih postopkih za vključitev pacienta v obravnavo fizioterapevt namesti KT
- izposoja algometra na Zdravstveni fakulteti (Maja Potočnik), meritve z algometrom opravi respiratorni fizioterapevt, na sliki telesa označi mesto največje bolečine

***PROTOKOL RESPIRATORNEGA FIZIOTERAPEVTA OB VKLJUČITVI:***

- bolnika, ki izpolnjuje kriterije za vključitev v raziskavo, se prijazno povabi v raziskavo: »da bomo lahko bolj natančno spremljali njegovo dihanje, ki je spremenjeno zaradi poškodbe reber«.
- obvesti se koordinatorja
- izvedba meritev in beleženje izmerjenih podatkov po protokolu
- izvedba dihalnih vaj (trebušno/preponske dihalne vaje)
- pri izbranem pacientu namestimo KT

**15 min po prvih meritvah:**

- izvedba dihalnih vaj
- izvedba vseh meritev in beleženje vseh podatkov po protokolu
- na raportu se poroča o vključitvi pacienta v raziskavo (evidenca vključenih bolnikov- Maja Potočnik)

**DAN 0:** *OB VKLJUČITVI:*

**1.tabela:**

| ***FVC*** |  |
| --- | --- |
| ***FEV1*** |  |
| ***PEF*** |  |
| ***FiO_2_ (označi %VM, pretok O_2_)*** |  |
| ***razlika v obsegu prsnega koša med vdihom in izdihom (cm)*** |  |
| ***VAS*** |  |
| ***MIP/ MEP*** |  |
| ***meritev z algometrom*** |  |
| meritev z myotonom |  |
| vrsta analgetika |  |
| dnevna poraba analgetikov |  |
| pH |  |
| pO_2_ |  |
| pCO_2_ |  |
| HCO_3_ |  |
| SaO_2_ |  |
| BE |  |
| ***pripombe respiratornega fizioterapevta*** |  |

- vrsta analgetikov in dnevna poraba (vpiše zdravnik)

**DAN 0:** *PO NAMESTITVI KT:*

**2.tabela:**

| ***FVC*** |  |
| --- | --- |
| ***FEV1*** |  |
| ***PEF*** |  |
| ***FiO_2_ (označi %VM, pretok O_2_)*** |  |
| ***razlika v obsegu prsnega koša med vdihom in izdihom (cm)*** |  |
| ***VAS*** |  |
| ***MIP/MEP*** |  |
| ***meritev z algometrom*** |  |
| meritev z myotonom |  |
| ***pripombe respiratornega fizioterapevta*** |  |

*PODATKI IZVEDBE:*

Izvajalci:	RFT:____________________________________	datum, ura:________

ZDRAVNIK: _____________________________ datum, ura:________

**2. DAN:**

**3.tabela:**

| ***FVC*** |  |
| --- | --- |
| ***FEV1*** |  |
| ***PEF*** |  |
| ***FiO_2_ (označi %VM, pretok O_2_)*** |  |
| ***razlika v obsegu prsnega koša med vdihom in izdihom (cm)*** |  |
| ***VAS*** |  |
| ***MIP/MEP*** |  |
| ***meritev z algometrom*** |  |
| meritev z myotonom |  |
| vrsta analgetika |  |
| dnevna poraba analgetikov |  |
| pH |  |
| pO_2_ |  |
| pCO_2_ |  |
| HCO_3_ |  |
| SaO_2_ |  |
| BE |  |
| ***pripombe respiratornega fizioterapevta*** |  |

- vrsta analgetikov in dnevna poraba (vpiše zdravnik)

*PODATKI IZVEDBE:*

Izvajalci:	RFT:____________________________________	datum, ura:________

ZDRAVNIK: _____________________________ datum, ura:________

**5. DAN:**

**4.tabela:**

| ***FVC*** |  |
| --- | --- |
| ***FEV1*** |  |
| ***PEF*** |  |
| ***FiO_2_ (označi %VM, pretok O_2_)*** |  |
| ***razlika v obsegu prsnega koša med vdihom in izdihom (cm)*** |  |
| ***VAS*** |  |
| ***MIP/MEP*** |  |
| ***meritev z algometrom*** |  |
| meritev z myotonom |  |
| vrsta analgetika |  |
| dnevna poraba analgetikov |  |
| pH |  |
| pO_2_ |  |
| pCO_2_ |  |
| HCO_3_ |  |
| SaO_2_ |  |
| BE |  |
| ***pripombe respiratornega fizioterapevta*** |  |

- vrsta analgetikov in dnevna poraba (vpiše zdravnik)

*PODATKI IZVEDBE:*

Izvajalci:	RFT:____________________________________	datum, ura:________

ZDRAVNIK: _____________________________ datum, ura:________

**10. DAN (po telefonu):**

**5.tabela:**

| VAS |  |
| --- | --- |
| vrsta analgetika |  |
| dnevna poraba analgetikov |  |
| vprašalnik o kvaliteti življenja |  |

Izvedel : ____________________________________	datum, ura:________

NAVODILA ZA APLIKACIJO ELASTIČNIH LEPILNIH TRAKOV

Pred aplikacijo poskrbimo za vsaj minimalen razteg tkiva (kože in podkožja) v področju poškodbe, v kolikor to ne poslabša bolečine. Širino in dolžino traku prilagodimo mestu poškodbe/okvare. Koža mora biti suha in čista. Po aplikaciji pacienta vedno vprašamo o občutku, v primeru poslabšanja bolečin, občutka utesnjenosti, srbenja, pojava rdečine, trakove odstranimo.

Slika 1: ligamentna tehnika s 50% raztegom v osrednjem delu traku nad in pod področjem poškodbe/okvare

Slika 2: ligamentna tehnika s 50% raztega prečno na prvi del aplikacije, medialno in lateralno od področja poškodbe/okvare

Slika 3: končni položaj aplikacije

Pripravila: Maja Frangež
